# Association between human leucocyte antigen subtypes and risk of end stage renal disease in Taiwanese: a retrospective study

**DOI:** 10.1186/s12882-015-0165-7

**Published:** 2015-10-30

**Authors:** Ciou-Sia Dai, Chen-Chung Chu, Shin-Fan Chen, Chiao-Yin Sun, Marie Lin, Chin-Chan Lee

**Affiliations:** 1 Department of Internal Medicine, Division of Nephrology, Chang Gung Memorial Hospital, Keelung, Taiwan; 2 Department of Medical Research, Mackay Memorial Hospital, Taipei, Taiwan; 3 Department of Bioscience Technology, Chung Yuan Christian University, Chung-Li, Taiwan; 4 Department of Nephrology, Chang Gung Memorial Hospital, Keelung, Taiwan

**Keywords:** ESRD, Human leukocyte antigen, Taiwan, Chronic kidney disease, HLA types

## Abstract

**Background:**

End stage renal disease (ESRD) is prevalent in Taiwan. Human leukocyte antigens (HLA) have been found to be associated with the pathogenesis of autoimmune diseases, allergies and inflammatory bowel diseases, and there are emerging evidences of correlations between HLA genotypes and renal diseases such as diabetic nephropathy, IgA nephropathy, and glomerulonephritis. The aim of this study is to investigate detailed HLA subtypes in a case-control study of Taiwanese individuals.

**Methods:**

The polymorphisms of HLA class I and II antigens in ESRD patients and a healthy control group were retrospectively analyzed. The information of 141 ESRD patients was obtained from the medical record of the Keelung branch of Chang Gung Memorial Hospital and was compared to the HLA type of a control group comprized of 190 healthy unrelated Taiwanese from one of our previous studies. In order to standardize the HLA designation of prior low-resolution typings with the more advanced DNA based typings, all HLA-A, −B and -DR were analyzed using a low resolution serologic equivalent.

**Results:**

The current work suggests that HLA-DR3 (odds ratio = 1.91, 95 % CI = 1.098–3.324, *P* = 0.024, Pc = 0.312) and HLA-DR11 (odds ratio = 2.06, 95 % CI = 1.133–3.761, *P* = 0.021, *P*c = 0.273) may represent susceptibility risk factors for the development of ESRD in Taiwanese individuals. On the other hand, HLA-DR8 (odds ratio = 0.47, 95 % CI = 0.236–0.920, *p* = 0.027. Pc = 0.351) may be a protective factor. HLA-A and -B antigens did not show any contribution of progression to ESRD. However, we note that the significance of all these findings is lost when the results are corrected for multiple comparisons according to Bonferroni. Further investigation with a larger group of patients and control is needed to resolve this issue.

**Conclusions:**

HLA typing might be a useful clinical method for screening patients with high risk of progression to ESRD.

## Background

The prevalence and incidence of chronic kidney disease (CKD) and end stage renal disease (ESRD) are high in Taiwan [1], and the morbidity associated with the ESRD has become a serious public health issue. One possible reason is that preventive care of CKD is low in the Taiwan [2] and the causes of CKD among Taiwanese are diverse, the most common being diabetes mellitus, hypertension, and glomerulonephritis [3]. It is worth noting that for about 48 % of early-stage and 25 % of late-stage CKD patients, the causes of the disease are not well defined [3]. Although no clear risk factors have been defined for these patients it is believed that their demography and proper access to medical care largely contribute to the lack of prevention and poor management of CKD. Presently, the screening of individuals without apparent symptoms or not at risks is not applied in Taiwan [4].

The HLA system belongs to the major histocompatibility complex (MHC) in humans and it is located on chromosome 6p21.3. HLA genes encode cell surface molecules specialized to present antigenic peptides to T-cell receptors. MHC molecules are divided into two main classes: MHC class I and II. The heavy chain of the class I molecule is encoded by genes at the HLA-A, HLA-B, and HLA-C loci, and class II MHC molecules are encoded by genes in the HLA-DP, HLA-DQ, or HLA-DR regions [5, 6]. Specific HLA types have been known to be associated with the pathogenesis of many autoimmune diseases, allergies, and inflammatory bowel disease [7–10]. The detection of specific HLA types has proven to be a valuable tool for the diagnosis or screening of ankylosing spondylitis, inflammatory bowel disease, and multiple sclerosis [11–13]. Several emerging studies have described significant correlations between HLA and some renal diseases such as diabetic nephropathy, IgA nephropathy, and glomerulonephritis [14–16]. However, specific HLA types associated with ESRD have not been well documented. In this study, HLA class I and II polymorphisms of ESRD patients were compared to a healthy control group in an effort to provide a better understanding of the etiology of this disease.

## Methods

### Study group*s*

This retrospective analysis uses data from 141 Taiwanese ESRD patients under the age of 50 years who were awaiting kidney transplantation between the years 2002 and 2013 at the Keelung branch of Chang Gung Memorial Hospital. General clinical characteristics, HLA typing, and causes of ESRD were obtained from the health records of the organ donation and transplantation office of the hospital. The control group included 190 unrelated healthy Taiwanese individuals from a previous study that we conducted at the Mackay Memorial Hospital in Taipei to investigate the association between HLA polymorphism and multibacillary leprosy [17]. All patient and control individuals were Taiwanese, descendant of early Minnan or Hakka Chinese from the Fukien and Kwangton provinces on the south-east-coast of China who settle in Taiwan in the last 400 years. Other studies have shown that, although Minnan and Hakka speak different Chinese dialects, they have a similar HLA profile [18, 19]. Allele frequencies of Minnan and Hakka in our previous study have been deposited in a worldwide database (http://www.allelefrequencies.net/). In this retrospective study, ethical approval was obtained by the institutional review board of medical ethics and the human body test committee at the Chang Gung Memorial Hospital (102-5322B).

### HLA typing

HLA typing was initially performed by serological method or DNA based typing method at the time of the onset of the disease. The 74 ESRD patients who entered the transplantation waiting list before year 2008 were typed by complement dependent cytotoxicity (CDC) testing method,whereas the 67 ESRD patients who enrolled after the year 2008 were typed by reverse line blot using the RELI™ SSO typing kit (Dynal Biotech, Bromborough, Wirral, UK). Finally, DNA-based typing results were converted to serologic designations according to the HLA dictionary 2008 [20].

### Statistical analysis

Antigen counts were obtained from the serologic data. Statistical analyses for the association between patient and control groups were performed by estimating the odds ratios (OR) and 95 % confidence intervals (95 % CI) using the approximation method of Woolf using GraphPadInStat version 3.0 (GraphPad Software, San Diego, CA). Two tailed *P* values were estimated by Fisher’s exact test. A *P* value less or equal to 0.05 was considered to be significant. Corrected *P* values (*P*c) were also calculated by multiplying the *P* values by the number of antigens represented in the samples (according to the Bonferroni’s correction).

## Results

### General characteristics of the study population

HLA polymorphism was analyzed to determine the differences between 190 healthy control individuals and 141 ESRD patients (Table 1). Most ESRD patients had unknown primary disease (*n* = 89, 63.2 %), and diabetes mellitus type 2 was the most common cause of ESRD (*n* = 30, 21.3 %).

**Table 1:** Baseline characteristics of the study population

Total	141
Male/Female	80 (56.7 %)/61 (43.3 %)
Mean age at the time of end stage renal disease	40 ± 12 y
Causes of end stage renal disease	
1. Diabetes mellitus	30 (21.3 %)
2. IgA nephropathy	9 (6.4 %)
3. Autosomal polycystic kidney disease	4 (2.8 %)
4. Focal segmental glomerulosclerosis	3 (2.1 %)
5. Minimal change disease	3 (2.1 %)
6. Rapidly progressive GN	1 (0.7 %)
7. Membranous nephropathy	1 (0.7 %)
8. Mesangioproliferative GN	1 (0.7 %)
9. Unknown	89 (63.2 %)

### Association of HLA-A and HLA-B antigens with ESRD

HLA class I analysis in patients and control (Table 2 and Table 3) revealed 13 HLA-A and 28 HLA-B antigens. The most common HLA-A locus antigens with antigen frequency greater than 10 % in the two groups were A11, A2, A24, and A33. Similarly, the most common HLA-B locus antigens were B60, B46, and B58. We note that HLA-A and -B antigens distribution in the two groups were similar and that no significant differences (odds ratio) were found between them.

**Table 2:** HLA-A antigen frequency among individuals with ESRD and healthy controls

	Patients *N* = 141		Control *N* = 190				
Antigens	Count	Antigen frequency	Count	Antigen frequency	Odds ratio	95 % confidence interval (95 % CI)	*P* value
A1	4	2.8 %	1	0.5 %	5.52	0.610–49.920	NS
A2	61	43.3 %	96	50.5 %	0.75	0.482–1.157	NS
A3	2	1.4 %			6.83	0.325–143.350	NS
A24	48	34.0 %	59	31.1 %	1.15	0.720–1.824	NS
A11	86	61.0 %	112	58.9 %	1.09	0.698–1.699	NS
A26	5	3.5 %	10	5.3 %	0.66	0.221–1.981	NS
A29	1	0.0 %	1	0.5 %	1.35	0.084–21.771	NS
A30	7	5.0 %	3	1.6 %	3.26	0.827–12.822	NS
A31	3	2.1 %	9	4.7 %	0.44	0.116–1.645	NS
A32			2	1.1 %	0.27	0.013–5.593	NS
A33	32	22.7 %	37	19.5 %	1.21	0.712–2.069	NS
A34	3	2.1 %			9.63	0.493–187.918	NS
A68			1	0.5 %	0.45	0.018–11.040	NS

*N* number, *NS* not significant

**Table 3:** HLA-B antigen frequency among individuals with ESRD and healthy controls

	Patients *N* = 141		Control *N* = 190				
Antigens	Count	Antigen frequency	Count	Antigen frequency	Odds ratio	95 % confidence interval (95 %CI)	*P* value
B13	25	17.7 %	34	17.9 %	0.99	0.560–1.748	NS
B18	1	0.7 %	1	0.5 %	1.35	0.083–21.771	NS
B27	12	8.5 %	15	7.9 %	1.09	0.491–2.397	NS
B35	10	7.1 %	10	5.3 %	1.37	0.556–3.397	NS
B37	2	1.4 %	1	0.5 %	2.72	0.244–30.292	NS
B38	11	7.8 %	16	8.4 %	0.92	0.413–2.049	NS
B39	10	7.1 %	8	4.2 %	1.74	0.667–4.520	NS
B44	1	0.7 %	2	1.1 %	0.67	0.060–7.479	NS
B46	28	19.9 %	46	24.2 %	0.78	0.456–1.318	NS
B48	7	5.0 %	5	2.6 %	1.93	0.601–6.221	NS
B51	10	7.1 %	21	11.1 %	0.61	0.278–1.349	NS
B52	2	1.4 %	2	1.1 %	1.35	0.188–9.720	NS
B54	10	7.1 %	15	7.9 %	0.89	0.388–2.046	NS
B55	8	5.7 %	13	6.8 %	0.82	0.330–2.033	NS
B56	5	3.5 %	3	1.6 %	2.29	0.539–9.753	NS
B58	34	24.1 %	37	19.5 %	1.31	0.776–2.226	NS
B60	46	32.6 %	66	34.7 %	0.91	0.573–1.444	NS
B61	16	11.3 %	16	8.4 %	1.39	0.671–2.889	NS
B62	13	9.2 %	18	9.5 %	0.97	0.459–2.053	NS
B67			1	0.5 %	0.45	0.018–11.040	NS
B7			2	1.1 %	0.27	0.013–5.593	NS
B71			2	1.1 %	0.27	0.013–5.593	NS
B75	17	12.1 %	21	11.1 %	1.10	0.556–2.178	NS
B76	2	1.4 %	1	0.5 %	2.72	0.244–30.292	NS
B70	1	0.7 %	0		4.07	0.165–100.598	NS
B57	1	0.7 %	0		4.07	0.165–100.598	NS
B81	1	0.7 %	0		4.07	0.165–100.598	NS
B40	1	0.7 %	0		4.07	0.165–100.598	NS

*N* number, *NS* not significant

### Association of HLA-DR antigens with ESRD

The combined HLA class II polymorphism revealed 13 DR antigens (Table 4) with HLA-DR9, DR4, DR11, DR12, DR15, DR8, DR3, DR14, and DR16 being the only antigens having a frequency greater than 10 %. ESRD disease assessment revealed positive associations with HLA-DR3 (odds ratio = 1.91, 95 % CI = 1.098–3.324, *P* = 0.024, *P*c = 0.312) and HLA-DR11 (odds ratio = 2.06, 95 % CI = 1.133–3.761, *P* = 0.021, *P*c = 0.273), and a negative association with HLA-DR8 (odds ratio = 0.47, 95 % CI = 0.236–0.920, *P* = 0.027,Pc = 0.351) (Table 4). We note that the significance of these associations is lost after establishing Bonferroni correction.

**Table 4:** HLA-DR antigen frequency among individuals with ESRD and healthy controls

	Patients *N* = 141		Control *N* = 190					
Antigens	Count	Antigen frequency	Count	Antigen frequency	Odds ratio	95 % confidence interval (95 % CI)	*P* value	Corrected *P* value
DR1			3	1.6 %	0.19	0.010–3.695	NS	
DR3	35	24.8 %	28	14.7 %	1.91	1.098–3.324	0.024	0.312
DR4	48	34.0 %	54	28.4 %	1.30	0.813–2.079	NS	
DR7	9	6.4 %	4	2.1 %	3.17	0.956–10.513	NS	
DR8	13	9.2 %	34	17.9 %	0.47	0.236–0.920	0.027	0.351
DR9	44	31.2 %	56	29.5 %	1.09	0.676–1.743	NS	
DR10	6	4.3 %	4	2.1 %	2.07	0.572–7.466	NS	
DR11	30	21.3 %	22	11.6 %	2.06	1.133–3.761	0.021	0.273
DR12	29	20.6 %	48	25.3 %	0.77	0.454–1.292	NS	
DR13	6	4.3 %	17	8.9 %	0.45	0.174–1.178	NS	
DR14	14	9.9 %	25	13.2 %	0.73	0.364–1.456	NS	
DR15	24	17.0 %	36	18.9 %	0.88	0.496–1.551	NS	
DR16	10	7.1 %	25	13.2 %	0.50	0.23–1.086	NS	

*N* number, *NS* not significant

Most ESRD patients had unknown etiology (*N* = 89, 63.2 %) and only 21.3 % (*N* = 30) were Type II diabetes mellitus patients. After exclusion of all DM patients (Table 5), HLA-DR3 (OR = 1.95, *P* = 0.031, *P*c = 0.403) and DR11 (OR = 2.11, *P* = 0.030, *P*c = 0.39) remained significantly associated to ESRD whereas HLA-DR8 showed protection to the disease (OR = 0.40, *P* = 0.026, *P*c = 0.338). In Brief associations of DR antigens with non-DM patients remained unchanged and further suggest that the association of DR3 and DR11 is not relevant to the presence or absence of DM.

**Table 5:** HLA-DR antigen frequency in healthy control and non-DM individuals with ESRD

	Patients (*N* = 111)		Control (*N* = 190)					
Antigens	Count	Antigen frequency	Count	Antigen frequency	Odd ratios	95 % confidence interval (95 % CI)	*P* value	*P*c value
DR1			3	1.60 %	0.24	0.012–4.694	NS	
DR3	28	25.20 %	28	14.70 %	1.95	1.085–3.510	0.031	0.403
DR4	36	32.40 %	54	28.40 %	1.21	0.728–2.008	NS	
DR7	8	7.20 %	4	2.10 %	3.61	1.062–12.284	0.036	0.468
DR8	9	8.10 %	34	17.90 %	0.4	0.186–0.880	0.026	0.338
DR9	35	31.50 %	56	29.50 %	1.1	0.663–1.831	NS	
DR10	5	4.50 %	4	2.10 %	2.19	0.577–8.346	NS	
DR11	24	21.60 %	22	11.60 %	2.11	1.118–3.970	0.03	0.39
DR12	26	23.40 %	48	25.30 %	0.9	0.523–1.565	NS	
DR13	5	4.50 %	17	8.90 %	0.48	0.172–1.340	NS	
DR14	11	9.90 %	25	13.20 %	0.73	0.3425–1.539	NS	
DR15	17	15.30 %	36	18.90 %	0.77	0.412–1.454	NS	
DR16	8	7.20 %	25	13.20 %	0.51	0.223–1.179	NS	

## Discussion

ESRD is a condition where patients are imperatively dependent on renal replacement in order to avoid life-threatening uremia [21]. The HLA system has been found to be associated with the pathogenesis of autoimmune diseases, inflammatory bowel disease, allergies and some renal diseases such as diabetic nephropathy, IgA nephropathy and glomerulonephritis. Identification and analysis of the HLA polymorphism in ESRD patients is not only important for the determination of a possible association of the disease with HLA, but is also an absolute requirement for the selection of an optimal kidney matching for transplantation in these patients [22].

In this study, HLA-DR3, and HLA-DR11 antigen frequencies in the ESRD patient group were significantly higher than in the control group (DR3: cases 24.8 vs control 14.7 %; DR11: case 21.3 vs control 11.6 %) with OR values of 1.91 (*P* = 0.024) and 2.06 (*P* = 0.021), respectively. On the other hand, HLA-DR8 was significantly lower in the ESRD patient group than in the control group (case 9.2 vs control 17.9 %; OR 0.47; *P* = 0.027). However, after Bonferroni’ correction, all corrected *P* values were greater than 0.05 (Pc > 0.05). Although the uncorrected P value may suggest type I error, we can estimate that a data set only three times larger would maintain significance after Bonferroni correction. Such reachable prospect justifies further analyses for confirmation of these results.

### HLA-DR3

Previously reported associations between HLA class I and II, and ESRD among patients with history of diabetes, hypertension, and various types of glomerulonephritis are summarized in Table 6 [16, 23–33]. These studies show that HLA-DR3 was significantly associated with membranous nephropathy in Chinese, French, British, Chilean, and North American [50,51,52], DR3 was also associated with the occurrence of diabetic nephropathy [24, 29, 30, 34, 35], and was protective against the occurrence of idiopathic IgA nephropathy [36]. In support to these studies, our results show that HLA-DR3 was increased in the ESRD group (patients 23 vs control 14 %) with an OR significant before Bonferroni correction.

**Table 6:** Review of systemic and kidney diseases associated with HLA type

Population	Study End point	Susceptibility		Protection		Reference
		MHC class I	MHC class II	MHC class I	MHC class II	
Taiwan	ESRD		DR3,DR11		DR8	*
Kuwaiti	ESRD	B8		A28	DR11	[42]
Saudi	ESRD		DQB1*03(8)	Cw2		[43]
	Glomerulonephritis					
China	Poor renal outcome of ANCA related vasculitis		DRB1*04:05, DPB1*0402			[33]
China	Cresentic GN in anti-GBM disease		DRB1*1501 DRB1*0404			[32]
Italy	Churg-Strauss syndrome with renal involvement		DRB*04			[28]
United States	Anti-GBM disease		DRB1*15 DRB1*04		DRB1*07	[23]
Taiwan	Lupus nephritis				DRB1*1202	[31]
Italy	Lupus nephritis		DRB1*1501, DQA1*0101		DQA1*0102	[26]
United States	IgA nephropathy	B27	DR1		DR2	[44]
Japan	IgA nephropathy		DR4			[14]
France	IgA nephropathy	B35				[45]
Europe	IgA nephropathy	Bw35				[46]
Netherland	Idiopathic IgA nephropathy	B35	DR5	B7,B8	DR2,DR3	[36]
China	IgA nephropathy		DR14, DR3		DR7	[47]
Sweden	IgA nephropathy		DR4			[16]
France	IgA nephropathy		DQB1*0301			[48]
Japan	IgA nephropathy	Bw35	DR4			[41]
Japan	IgA nephropathy		DQw4			[49]
France	Membranous GN		DR3			[50]
Taiwan	Membranous GN		DR3			[51]
UnitedStates	Membranous GN		DR3, DR5		DR7	[52]
Netherland	Idiopathic MN	B8	DR3			[53]
South Africa	HBV- associated membranous GN in children		DQB1*0603			[54]
Korea	HBV associated GN		DR2, DRB1*15:01, DRB1*15:02		DRB1*1302, DQB1*0402, DQB1*0604	[27]
United States	Heroin- associated nephropathy	Bw53				[55]
United States	Hypertensive renal failure	B35	DR3	A1, B8		[56]
Brazil	Idiopathic FSGS		DR4			[25]
	Systemic diseases					
Mexico	Type 2 diabetes mellitus with ESRD		DRB1*1502 DQB1*0501		DRB1*0407	[34]
London	Early diabetic nephropathy	A2				[24]
Turkey	Amyloidosis and diabetic nephropathy	B58	DR*03			[30]
United States	Diabetic nephropathy				DRB1*04	[29]
Canada	Diabetes related ESRD in ≤ 50y	A2	DR4, DR8			[15]
Egypt	Diabetic nephropathy	A2, B8	DRB1*3, DRB1*11			[35]

*DESRD* diabetic related end stage renal disease

*y* years

*Results of this study

We further note that HLA-DR11 (Table 6) was associated with diabetic nephropathy in Egyptian population [35] and other diseases such as celiac disease, rheumatic heart disease, and cancer [37–40]. In our study, the occurrence of HLA-DR11 was significantly higher in the ESRD group. Similarly to HLA-DR3, the significance of HLA-DR11 was lost after Bonferroni correction. Again, while suggesting that a larger data set is required to support to these results, one should be aware that DR3 and DR11 are potentially valuable predictors for evaluating the risk of ESRD in the Taiwanese population.

HLA-DR8 has been associated with the prevalence of DESRD in individuals under 50 years [15]. In our study, the presence of HLA-DR8 was significantly lower in patients than in the control group and may have a protective influence against the incidence of ESRD.

### HLA-DR4

In previous studies, HLA-DR4 has been associated with immune complex-mediated rapidly progressive glomerulonephritis in populations from China, Italy and the USA [23, 28, 32, 33] and showed strong association with the occurrence of IgA nephropathy in the Japanese and with idiopathic focal sclerosing glomerulosclerosis in the Brazillian population [25, 41]. Individuals with HLA-DR4 were also susceptible to DESRD in patients under 50 years old from Canada [15], but was protective from diabetic nephropathy in the US and Mexican populations [29, 34]. In this study, the frequency of HLA-DR4 is higher in patients (34 %) than it in the control group (28 %), but this difference was not significant.

In brief, this study reports two HLA antigens (DR3 and DR11) that showed significant associations with the risk of progression to ESRD. However, both control and disease study groups were too small to sustain the significance after Bonferonni correction. However, although our data set was small, we find that after stratification of our data set for non-T2DM the same level of significance was obtained suggesting that DR3 and DR11 association to ESRD may be independent to any specific disease group.

To the best of our knowledge, this analysis is the first case–control study to analyze the association between the HLA polymorphisms and the risk to develop ESRD in a Taiwanese population. Further, the analysis showed several significant DR associations with ESRD indicating that HLA class II polymorphism might be a useful clinical tool for screening patients with high risk of ESRD and constitute sufficient motivating elements to undertake early preventive measures in the management of ESRD.

## Conclusion

HLA polymorphism might be a useful clinical tool for screening patients with high risk of ESRD. This analysis used small population case and control data set and warrant further study to confirm these results.

## Abbreviations

ESRD: End stage renal disease
HLA: Human leukocyte antigen
